# Presence and Characteristics of Behavioral and Psychological Symptoms in Subacute Stroke Patients with Cognitive Impairment

**DOI:** 10.1155/2023/6636217

**Published:** 2023-12-28

**Authors:** Daisuke Ito, Naoki Mori, Ayaka Shimizu, Ayako Narita, Sachiko Sakata, Kaoru Honaga, Kunitsugu Kondo, Yohei Otaka

**Affiliations:** ^1^Department of Rehabilitation Medicine, Keio University School of Medicine, Tokyo, Japan; ^2^Department of Rehabilitation Medicine, Tokyo Bay Rehabilitation Hospital, Chiba, Japan; ^3^Department of Well-Being and Rehabilitation, School of Medicine, Fujita Health University, Aichi, Japan; ^4^Department of Rehabilitation Medicine, Juntendo University Graduate School of Medicine, Tokyo, Japan; ^5^Department of Rehabilitation Medicine I, School of Medicine, Fujita Health University, Aichi, Japan

## Abstract

This retrospective cross-sectional study is aimed at investigating the prevalence and characteristics of behavioral and psychological symptoms (BPS) in subacute stroke patients with cognitive impairment. The Neuropsychiatric Inventory-Questionnaire (NPI-Q) was used to assess BPS. A total of 358 consecutive patients with first-ever stroke admitted to rehabilitation wards and with Mini-Mental State Examination (MMSE) scores < 24 on admission were included. BPS was defined as a total NPI-Q Severity or Distress score ≥ 1. Differences between the severity and presence of BPS among patients with severe cognitive impairment (MMSE scores 0–17) and those with mild cognitive impairment (MMSE scores 18–23) were analyzed using the Mann–Whitney *U* test and chi-squared test, respectively. Eighty-one patients (mean (standard deviation) age, 73.5 (13.1) years) were enrolled for analysis. BPS were observed in 69.1% and 74.1% of patients when assessed with NPI-Q Severity and NPI-Q Distress, respectively. The most frequently observed BPS was apathy, followed by depression (approximately 44% and 40%, respectively). The severity and frequency of delusions, euphoria, apathy, and disinhibition were significantly higher in the severe cognitive impairment group than in the mild cognitive impairment group. However, the severity, distress, and frequency of depression were not dependent on the severity of cognitive impairment. The presence of BPS, especially apathy and depression, in subacute stroke patients with cognitive impairment is high. The severity and frequency of some BPS are higher in patients with severe cognitive impairment than in those with mild cognitive impairment. However, depression is highly prevalent among the patients regardless of the severity of cognitive impairment.

## 1. Introduction

Neuropsychiatric symptoms are core features of dementia [1]. Behavioral and psychological symptoms (BPS), which are a combination of various symptoms, are some of the most common neuropsychiatric symptoms [2]. BPS have been reported to be associated with caregiver burden [3, 4], caregiver depression [4], long-term hospitalization [5], and increased healthcare costs [5].

Alzheimer's disease and vascular dementia are the diseases very frequently associated with BPS [6]. A previous study has reported that the prevalence of BPS was very high, reaching 100% in AD and VD [7]. Regarding differences in each symptom, a systematic review revealed that the prevalence of delusions, anxiety, apathy, irritability, elation, mania, and aberrant motor behavior in patients with Alzheimer's disease was more significant than that in patients with vascular dementia, whereas disinhibition was more significant in patients with vascular dementia than in those with Alzheimer's disease [6]. Thus, BPS have different characteristics depending on the type of dementia [8]. However, most previous studies on BPS have focused on Alzheimer's disease, and the limited knowledge can be obtained for vascular cognitive impairment (VCI), which is now preferred to as vascular dementia and includes a broad spectrum of cognitive disorders, from mild cognitive impairment to vascular dementia caused by ischemic or hemorrhagic stroke [9]. Only a few studies examined the prevalence of BPS in chronic phase VCI and reported to be approximately 90% [10–12]. Especially, there are limited reports on VCI in the subacute phase [13], when patients improve their function through rehabilitation. Because the prevalence of VCI in the subacute phase has been reported to be substantially high, ranging 26–76% [14–17], and poststroke BPS are associated with poor functional outcomes [18] and increased risk of cognitive decline [19], it is necessary to thoroughly investigate BPS among patients with subacute stroke. Furthermore, changes in BPS according to the severity of cognitive impairment, even in patients with the same disease [8], including VCI [10], require clarification. Thus, this study is aimed at elucidating the prevalence and characteristics of BPS in patients with subacute stroke who showed cognitive impairment and examining the differences in the BPS of patients according to the severity of cognitive impairment.

## 2. Methods

### 2.1. Study Design and Participants

**Figure 1:**
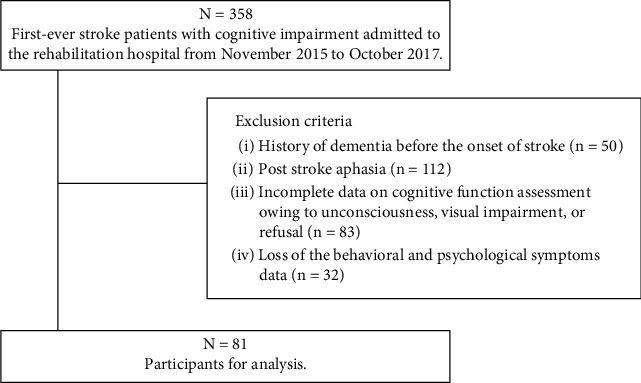
Flow diagram of study participants.

This retrospective cross-sectional study was performed according to STROBE guidelines. A total of 358 consecutive patients with subacute stroke, admitted to Tokyo Bay Rehabilitation Hospital between November 2015 and October 2017, were selected for this study. The inclusion criteria were first-ever stroke and a Mini-Mental State Examination (MMSE) score of less than 24 on admission. The exclusion criteria were a history of dementia before the onset of stroke (*n* = 50), a history of psychiatric symptoms (such as depression or bipolar disorder) before the onset of stroke (*n* = 5), poststroke aphasia (*n* = 111), incomplete data on cognitive function assessment owing to unconsciousness, visual impairment, or refusal (*n* = 79), and partially or completely missing BPS data (*n* = 32). Finally, 81 patients were included in the study (Figure 1). The protocol for this study was reviewed and approved by the Institutional Ethics Committee of the Tokyo Bay Rehabilitation Hospital (#148).

### 2.2. Data Collection

The basic patient characteristics, including age, sex, duration from stroke onset to admission to the rehabilitation ward, type of stroke, Brunnstrom recovery stage of motor function [20], MMSE score [21], and Functional Independence Measure [22] at admission, were obtained from medical records. These measurements have already established validity and reliability in stroke patients [23–25]. Measures for BPS evaluation were also obtained.

### 2.3. Brunnstrom Recovery Stage

Motor function was assessed using the Brunnstrom recovery stage of motor function [20], which consists of three tests for the upper extremity, hand, and lower extremity. Each test was rated on a 6-grade ordinal scale ranging from stage I (flaccid, no voluntary movement) to stage VI (isolated joint movement).

### 2.4. Mini-Mental State Examination

MMSE is a questionnaire for evaluating cognitive function [21]. It consists of 11 items as follows (maximum score of each item): orientation to time (5), orientation to place (5), registration of three words (3), attention and calculation (serial sevens or spelling) (5), recall (3), naming (2), repetition (1), comprehension of verbal (3), comprehension of written (1), writing (1), and construction (1). The maximum score is 30 points, with a higher score representing greater cognitive function.

### 2.5. Functional Independence Measure

Functional Independence Measure is an observational evaluation tool for functional disability [22]. The FIM consists of 13 motor subscales and five cognitive subscales. The motor subscales consist of the following four categories: self-care (eating, grooming, bathing, dressing-upper body, dressing-lower body, and toileting), sphincter control (bladder management and bowel management), transfers (bed/chair/wheelchair, toilet, and tub/shower), and locomotion (walk/wheelchair and stairs). The cognitive subscales consist of two categories: communication (comprehension and expression) and social cognition (social interaction, problem-solving, and memory). Each item has a 7-grade scale ranging from 1 (total assistance or not testable) to 7 (complete independence) points. The total score is 18–126 points, 13–91 points, and 5–35 points for the total score, motor score, and cognitive score, respectively, with a higher score representing greater functional independence.

### 2.6. Neuropsychiatric Inventory-Questionnaire

The Neuropsychiatric Inventory-Questionnaire (NPI-Q) is an observational evaluation tool used for measuring the severity of BPS and the burden of care associated with BPS [26]. It consists of the original 10 items (delusions, hallucinations, agitation, depression, anxiety, euphoria, apathy, disinhibition, irritability, and aberrant motor behavior) plus items on eating abnormalities and sleep disturbances. Each item used for analyzing severity and distress is scored using four grades (0, not at all; 1, mild; 2, moderate; and 3, severe) and six grades (0, not at all; 1, minimal; 2, mild; 3, moderate; 4, severe; and 5, extreme or very severe), respectively. The validity and reliability of this tool [27], and its Japanese version [28], for the assessment of patients with stroke have been demonstrated previously. We used the original 10 items on the scale in this study. The total score for severity and burden of care ranges from 0 to 30 and 0 to 50, respectively, with larger scores indicating worse symptoms.

NPI-Q scores were evaluated at approximately two weeks after hospitalization. Occupational therapists evaluated the NPI-Q Severity scores, whereas nurses evaluated the NPI-Q Distress scores. The primary outcome was the total NPI-Q score and the presence of BPS. The secondary outcome was NPI-Q subitem scores and the presence of each symptom.

### 2.7. Analysis

Descriptive analyses of the NPI-Q scores were performed. The presence of BPS and the frequency of each symptom (≥1 point for each item) were calculated. BPS was defined as total NPI-Q score of ≥1 point. To examine the differences in BPS according to the severity of cognitive impairment, we classified the patients into two groups: severe cognitive impairment group (MMSE scores, 0–17) and mild cognitive impairment group (MMSE scores, 18–23) [29]. The normality of continuous variables was assessed using the normal Q-Q plot. The total NPI-Q scores, each item of the NPI-Q scores, and the presence of the symptom (defined as scoring ≥ 1 point in the item) were compared between the two groups using the chi-squared test, unpaired *t*-test, or Mann–Whitney *U* test, as appropriate. All statistical analyses were performed using IBM SPSS Statistics (version 27.0; IBM, Tokyo, Japan). *P* values < 0.05 were considered statistically significant.

## 3. Results

The characteristics of the study participants are listed in Table 1. The mean age (standard deviation (SD)) of the participants was 73.5 (13.1) years. The mean number of male participants was 35. The mean (SD) MMSE score on admission was 17.3 (4.7).

The presence of BPS and the NPI-Q scores are shown in Table 2. The mean (SD) total NPI-Q Severity score was 3.3 (3.9). The presence of BPS, which was defined as a total NPI-Q Severity score ≥ 1, was 69.1% (*n* = 56/81). Apathy was the most frequently reported NPI-Q Severity symptom (37 patients (45.7%)), followed by depression (33 patients (40.7%)) and anxiety (31 patients (38.3%)). Regarding the NPI-Q Distress score, the mean (SD) total score was 3.5 (5.2). The presence of distress in care, defined as a total NPI-Q Distress score ≥ 1, was 74.1% (*n* = 60/81). Apathy was also the most frequently reported NPI-Q Distress symptom (34 patients (42.0%)), followed by depression (32 patients (39.5%)) and anxiety (28 patients (34.6%)).

The comparison of the items and total score of the NPI-Q Severity scale between the severe cognitive impairment and mild cognitive impairment groups is presented in Table 3. The mean (SD) total NPI-Q Severity score in the severe cognitive impairment group and the mild cognitive impairment group was 4.2 (3.9) and 2.5 (3.8), respectively. The scores of the severe cognitive impairment group were significantly higher than those of the mild cognitive impairment group (*P* = 0.011). The presence of BPS in the severe cognitive impairment group and the mild cognitive impairment group was 83.3% and 57.8%, respectively (*P* = 0.081). With respect to each item of the NPI-Q Severity scale, the scores and percentages of participants that scored ≥1 point in the delusions, euphoria, apathy, and disinhibition items were significantly higher in the severe cognitive impairment group than in the mild cognitive impairment group (*P* < 0.05).

The comparison of the items and total scores of the NPI-Q Distress scale between the severe cognitive impairment and mild cognitive impairment groups is presented in Table 4. The mean (SD) total NPI-Q Distress score in the severe cognitive impairment group and the mild cognitive impairment group was 5.3 (6.6) and 2.1 (3.0), respectively. The scores of the severe cognitive impairment group were significantly higher than those of the mild cognitive impairment group (*P* = 0.005). The presence of distress in care in the severe cognitive impairment group and mild cognitive impairment group was 86.1% and 64.4%, respectively (*P* = 0.177). In the case of each item of the NPI-Q Distress scale, the scores and percentages of the participants that scored ≥1 point in the delusions, agitation, anxiety, apathy, and disinhibition items were significantly higher in the severe cognitive impairment group than in the mild cognitive impairment group (*P* < 0.05).

## 4. Discussion

This study investigated the BPS of subacute stroke patients with cognitive impairment who were admitted to rehabilitation wards. BPS were observed in approximately 70% of the participants. The most frequently observed symptom was apathy, followed by depression. The severity and frequency of delusions, euphoria, apathy, and disinhibition were significantly higher in the severe cognitive impairment group than in the mild cognitive impairment group. Similarly, the frequency of distress due to delusions, agitation, anxiety, apathy, and disinhibition was significantly higher in the severe cognitive impairment group than in the mild cognitive impairment group. In contrast, depression did not differ between the groups.

In the present study, the presence of BPS in subacute stroke patients with cognitive impairment was very high. To our knowledge, this is the first report of the presence of BPS in patients with subacute stroke admitted to rehabilitation wards. In the previous studies of chronic VCI, approximately 90% of the patients had at least one behavioral and psychological symptom [10–12], which is consistent with our findings. In addition, apathy and depression were frequently observed in these previous studies (approximately 44% and 40%, respectively). Apathy and depression are the most common neuropsychiatric poststroke symptoms [30]. Systematic reviews have shown that the pooled prevalence of apathy and depression among patients with stroke is 36.3% [31] and 33% [32], respectively. These findings are consistent with those of the present study. Thus, we suggest that subacute stroke patients with cognitive impairment already have a high presence of BPS on admission to rehabilitation wards.

Although the presence of apathy and depression in the present study was high, their characteristics differed significantly according to the severity of cognitive impairment. The severity, distress, and frequency of apathy were significantly higher in the severe cognitive impairment group than in the mild cognitive impairment group. Similarly, the severity or distress scores for several symptoms, such as delusions, agitation, anxiety, euphoria, and disinhibition, were significantly higher in the severe cognitive impairment group than in the mild cognitive impairment group. Other symptoms (hallucinations, irritability, and motor abnormalities) showed the same trend; however, the differences were not significant (*P* ≥ 0.05). A systematic review indicated that many BPS, including apathy, are exacerbated by the severity of cognitive impairment [6]. The results of the present study, which indicated an association between the severity of cognitive impairment and BPS, are consistent with these previous reports. The results of the present study also indicated that the relationship between depression and the severity of cognitive impairment was very different from those of the other BPS. Depression was the only symptom that showed higher frequency in the mild cognitive impairment group than in the severe cognitive impairment group. A previous study of patients in the chronic phase of stroke indicated that depression occurred irrespective of the severity of cognitive impairment [10]. Thus, depression may be an independent symptom rather than a symptom associated with cognitive impairment.

Considering the high severity, distress, and presence of BPS observed in the present study, stroke patients with cognitive impairment need to be managed for BPS from an early phase. Apathy and depression, both of which were highly prevalent in the present study, are known to interfere with functional recovery during rehabilitation [33]. Furthermore, apathy and depression are treated differently; thus, it is necessary to identify and provide appropriate treatment for each symptom. Poststroke depression can be treated using medications and other treatments [34]. In contrast, there are only a few high-quality treatments for apathy [35]. However, considering the association between apathy and the severity of cognitive impairment and the fact that patients in the subacute phase of stroke who are admitted to rehabilitation wards show improvements in cognitive impairment [36], intensive rehabilitation has a potential key role in alleviating apathy.

This study has several limitations. First, we assessed cognitive function with the MMSE; thus, patients with impaired consciousness or aphasia, whose cognitive function could not be assessed by MMSE, were excluded. Even patients with aphasia might possess BPS associated with cognitive impairment. Future studies using nonverbal cognitive assessments will explore the presence of BPS in a wider range of patients. Second, because this study focused on the presence of BPS, factors that were potentially associated with BPS, such as medications, stroke type, location and size of lesions, and dominant hemisphere, were not included in the analysis. Including those factors in the analysis would provide deeper understanding of the occurrence of BPS. Finally, this was a single-center, cross-sectional study. Thus, the generalizability of our results is limited. Despite these limitations, the present study is valuable in that it provides valuable information regarding the presence of BPS in subacute stroke patients and its characteristics according to the severity of cognitive impairment. Future multicenter prospective studies are needed to reveal longitudinal changes in BPS.

## 5. Conclusion

BPS are highly prevalent in subacute stroke patients with cognitive impairment who are admitted to rehabilitation wards. Apathy and depression are frequently observed in subacute stroke patients with cognitive impairment. Since apathy and depression are treated differently, it is essential to identify them in the early phase of stroke to enable timely provision of appropriate treatment.

## Figures and Tables

**Table 1:** Characteristics of the study participants.

Variables	Overall (*n* = 81)	MMSE < 18 (*n* = 36)	MMSE 18–23 (*n* = 45)
Age, year	73.5 (13.1)	74.1 (12.9)	73.0 (13.3)
Sex, male/female	35/46	15/21	20/25
Duration from onset of stroke, days	41.5 (17.1)	44.2 (21.6)	39.3 (12.0)
Type of stroke, infarction/hemorrhage/subarachnoid hemorrhage	43/26/12	19/10/7	24/16/5
Side of paresis, right/left/others	25/49/7	9/21/6	16/28/1
Brunnstrom recovery stage			
Upper extremity	3.6 (2.0)	3.2 (1.9)	3.9 (2.0)
Finger	3.6 (2.0)	3.3 (1.9)	3.8 (2.0)
Lower extremity	3.8 (1.9)	3.5 (1.9)	4.1 (1.9)
MMSE	17.3 (4.7)	13.0 (3.4)	20.8 (1.8)
Functional Independence Measure			
Motor score	34.4 (19.6)	27.7 (17.1)	39.7 (19.9)
Cognitive score	19.5 (6.1)	16.4 (4.8)	22.0 (5.8)
Total score	53.9 (23.1)	44.1 (19.4)	61.7 (22.8)

Values are presented as number or mean (standard deviation). MMSE: Mini-Mental State Examination.

**Table 2:** Neuropsychiatric Inventory-Questionnaire scores of subacute stroke patients with cognitive impairment.

Items	Severity		Distress	
	Number of patients according to score [0, 1, 2, 3]	Presence (% of scores ≥ 1)	Number of patients according to score [0, 1, 2, 3, 4, 5]	Presence (% of scores ≥ 1)
Delusions	[59, 13, 6, 3]	27.2	[61, 10, 5, 3, 2, 0]	24.7
Hallucinations	[74, 5, 0, 2]	8.6	[75, 5, 0, 0, 1, 0]	7.4
Agitation	[62, 14, 3, 2]	23.5	[59, 13, 4, 3, 1, 1]	27.2
Depression	[48, 27, 5, 1]	40.7	[49, 23, 6, 1, 2, 0]	39.5
Anxiety	[50, 25, 5, 1]	38.3	[53, 20, 8, 0, 0, 0]	34.6
Euphoria	[70, 7, 4, 0]	13.6	[70, 8, 3, 0, 0, 0]	13.6
Apathy	[44, 23, 9, 5]	45.7	[47, 25, 7, 1, 1, 0]	42.0
Disinhibition	[68, 9, 4, 0]	16.0	[69, 8, 2, 1, 0, 1]	14.8
Irritability	[69, 9, 2, 1]	14.8	[69, 8, 0, 2, 0, 2]	14.8
Aberrant motor behavior	[72, 7, 1, 1]	11.1	[72, 6, 1, 1, 0, 1]	11.1
Total score^∗^				
Mean (SD)	3.3 (3.9)	69.1	3.5 (5.2)	74.1
Median (IQR)	2 (0–5)		2 (0–4)	

Values are presented as number, percentage, mean (SD), or median (IQR). IQR: interquartile range; SD: standard deviation. ^∗^Total scores of Neuropsychiatric Inventory-Questionnaire range 0 to 30 and 0 to 50 in severity and distress, respectively, with higher scores indicating severer symptom.

**Table 3:** Comparison of Neuropsychiatric Inventory-Questionnaire Severity scores of patients with severe (MMSE < 18) and mild cognitive impairment (MMSE 18–23).

Items	Number of patients according to score [0, 1, 2, 3]		*P* value	Presence (% of scores ≥ 1)		*P* value
	MMSE < 18 (*n* = 36)	MMSE 18–23 (*n* = 45)		MMSE < 18 (*n* = 36)	MMSE 18–23 (*n* = 45)	
Delusions	[2, 5, 8, 21]	[38, 5, 1, 1]	0.008	41.7	15.6	0.009
Hallucinations	[32, 3, 0, 1]	[42, 2, 0, 1]	0.489	11.1	6.7	0.694
Agitation	[24, 10, 0, 2]	[38, 4, 3, 0]	0.080	33.3	15.6	0.061
Depression	[23,11, 2, 0]	[1, 3, 16, 25]	0.415	36.1	44.4	0.448
Anxiety	[19, 15, 2, 0]	[1, 3, 10, 31]	0.225	47.2	31.1	0.138
Euphoria	[27, 6, 3, 0]	[43, 1, 1, 0]	0.008	25.0	4.4	0.010
Apathy	[2, 5, 14, 15]	[3, 4, 8, 30]	0.038	61.1	33.3	0.013
Disinhibition	[26, 8, 2, 0]	[42, 1, 2, 0]	0.015	27.8	6.7	0.010
Irritability	[1, 6, 28]	[41, 3, 1, 0]	0.095	22.2	8.9	0.093
Aberrant motor behavior	[31, 4, 1, 0]	[41, 3, 0, 1]	0.491	13.9	8.9	0.501
Total score^∗^						
Mean (SD)	4.2 (3.9)	2.5 (3.8)	0.011	83.3	57.8	0.081
Median (IQR)	2.5 (1–6)	1 (0–3)				

Values are presented as number, percentage, mean (SD), or median (IQR). IQR: interquartile range; MMSE: Mini-Mental State Examination; SD: standard deviation. ^∗^Total scores of Neuropsychiatric Inventory-Questionnaire Severity range 0 to 30, with higher scores indicating severer symptom.

**Table 4:** Comparison of Neuropsychiatric Inventory-Questionnaire Distress scores of patients with severe (MMSE < 18) and mild cognitive impairment (MMSE 18–23).

Items	Number of patients according to score [0, 1, 2, 3, 4, 5]		*P* value	Presence (% of scores ≥ 1)		*P* value
	MMSE < 18 (*n* = 36)	MMSE 18–23 (*n* = 45)		MMSE < 18 (*n* = 36)	MMSE 18–23 (*n* = 45)	
Delusions	[22, 5, 5, 3, 1, 0]	[39, 5, 0, 0, 1, 0]	0.005	38.9	13.3	0.008
Hallucinations	[32, 3, 0, 0, 1, 0]	[43, 2, 0, 0, 0, 0]	0.249	11.1	4.4	0.399
Agitation	[1, 2, 9, 21]	[38, 4, 2, 1, 0, 0]	0.009	41.7	15.6	0.009
Depression	[23, 8, 3, 0, 2, 0]	[26, 15, 3, 1, 0, 0]	0.780	36.1	42.2	0.576
Anxiety	[18, 12, 6, 0, 0, 0]	[35, 8, 2, 0, 0, 0]	0.007	50.0	22.2	0.009
Euphoria	[28, 7, 1, 0, 0, 0]	[42, 1, 2, 0, 0, 0]	0.056	22.2	6.7	0.054
Apathy	[16, 15, 4, 0, 1, 0]	[31, 10, 3, 1, 0, 0]	0.037	55.6	31.1	0.027
Disinhibition	[26, 7, 1, 1, 0, 1]	[43, 1, 1, 0, 0, 0]	0.004	27.8	4.4	0.003
Irritability	[28, 5, 0, 1, 0, 2]	[41, 3, 0, 1, 0, 0]	0.087	22.2	8.9	0.093
Aberrant motor behavior	[30, 4, 1, 0, 0, 1]	[42, 2, 0, 1, 0, 0]	0.158	16.7	6.7	0.155
Total score^∗^						
Mean (SD)	5.3 (6.6)	2.1 (3.0)	0.005	86.1	64.4	0.177
Median (IQR)	3 (1–7)	2 (0–3)				

Values are presented as number, percentage, mean (SD), or median (IQR). IQR: interquartile range; MMSE: Mini-Mental State Examination; SD: standard deviation. ^∗^Total scores of Neuropsychiatric Inventory-Questionnaire Distress range 0 to 50, with higher scores indicating severer symptom.

## Data Availability

The datasets used and/or analyzed during the current study are available from the corresponding author on reasonable request.

## Supplementary Materials

Supplementary Materials STROBE Statement—checklist of items that should be included in reports of observational studies.
